# The Effect of Minimalist Versus Motion Control Shoes on Patellofemoral Joint Forces in Adolescents With Patellofemoral Pain During Running: A Randomized Crossover Study

**DOI:** 10.1177/03635465261443316

**Published:** 2026-05-16

**Authors:** Samual A. Kayll, Rana S. Hinman, Adam L. Bryant, Kim L. Bennell, Patrick L. Rowe, Pei Wei Chi, Scott Starkey, Fiona McManus, David Saxby, Kade L. Paterson

**Affiliations:** * Centre for Health, Exercise and Sports Medicine, Department of Physiotherapy, Melbourne School of Health Sciences, The University of Melbourne, Parkville, Australia; † Biostatistics Unit, Centre for Epidemiology and Biostatistics, Melbourne School of Population and Global Health, The University of Melbourne, Carlton, Victoria, Australia; ‡ Methods and Implementation Support for Clinical and Health Research Hub, Faculty of Medicine, Dentistry and Health Sciences, The University of Melbourne, Carlton, Victoria, Australia; § Australian Centre for Precision Health and Technology, Griffith University, Gold Coast, Queensland, Australia; Investigation performed at the Centre for Health, Exercise and Sports Medicine, Department of Physiotherapy, Melbourne School of Health Sciences, The University of Melbourne, Parkville, Australia

**Keywords:** adolescent, footwear, force, load, running, patellofemoral joint, patellofemoral pain, shoes

## Abstract

**Background:**

High joint forces can contribute to the pathogenesis of patellofemoral pain in adolescents. Minimalist shoes may reduce patellofemoral joint forces during running compared with commonly worn motion-control shoes.

**Hypothesis:**

Patellofemoral joint, lateral patella, and quadriceps forces will be lower, while gastrocnemius forces will be higher when adolescents run in minimalist shoes compared with motion control shoes.

**Study Design:**

Cross-sectional study; Level of evidence, 2.

**Methods:**

This within-session 2-period randomized crossover study was conducted from March 2023 to September 2024. A total of 51 physically active adolescents aged between 12 and 19 years (mean age, 16.9 ± 2 years) (23 female adolescents [45%]) with patellofemoral pain were recruited from the general population. Kinematic, kinetic, and electromyography (EMG) data were collected during overground running in minimalist and motion-control shoes, with testing randomized within the same session. Patellofemoral joint force was measured during the stance phase of running using an EMG-informed neuromusculoskeletal model. The primary outcome was the resultant patellofemoral joint force (N). Secondary outcomes included lateral patellar force (defined as the peak force acting on the patella in the frontal plane) and quadriceps and gastrocnemius muscle forces (N). All outcomes were analyzed at peak using paired *t* tests.

**Results:**

Compared with the motion control shoes, running in the minimalist shoes reduced peak resultant patellofemoral joint force by 7.5% (mean difference [MD], 363.2 N [95% CI, 666.8-59.5]; *P* = .02), reduced peak lateral patellar force by 7.8% (MD, 202.7 N [95% CI, 379-26.4]), and increased peak gastrocnemius muscle forces by 26.6% (MD, 449.3 N [95% CI, 298.6-560]). The minimalist shoe did not meaningfully alter quadriceps muscle forces compared with the motion-control shoe.

**Conclusion:**

As hypothesized, running in minimalist shoes reduced peak resultant patellofemoral joint and lateral patellar forces compared with running in motion-control shoes, while gastrocnemius forces were increased. Running in minimalist shoes did not meaningfully alter quadriceps muscle forces compared with motion-control shoes.

Patellofemoral (or kneecap) pain is most commonly seen in adolescents.^48^ Of those diagnosed with patellofemoral pain, 71% will reduce physical activity within 2 years^41^ and nearly half will have symptoms into adulthood.^17^ Patellofemoral pain is defined as pain at or around the patella during activities that increase force within the patellofemoral joint, such as running.^5,57^ High patellofemoral joint force increases patellar metabolic activity and water content (increasing intraosseous pressure) within the highly innervated subchondral bone, stimulating pressure-sensitive nociceptors, and producing pain.^7,16^ Importantly, the rapid growth of bone during adolescence precedes cartilage adaptation, making the subchondral bone particularly vulnerable to high forces.^53^ Reducing patellofemoral force during sporting activities may be a worthwhile strategy to minimize adolescent patellofemoral pain and prevent the potential negative consequences of inactivity.

Footwear can influence patellofemoral joint forces during running in adolescents. A substantial proportion of adolescents wear motion-control shoes (ie, stiff shoes with medial support designed to reduce the magnitude and/or rate of pronation)^24,27^ while running.^10^ However, running in motion-control shoes may increase patellofemoral joint contact force relative to minimalist shoes (ie, lightweight shoes with thin flexible soles that do not possess any motion control properties)^12^ (Figure 1). This may contribute to the high prevalence and long-term persistence of patellofemoral pain in adolescents.

**Figure 1.**
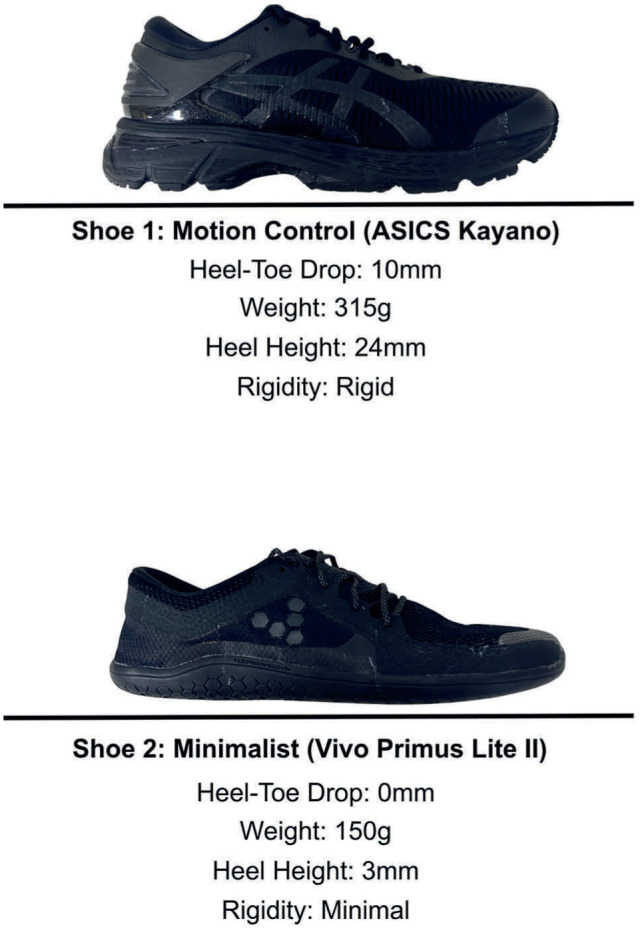
The motion control (top) and minimalist (bottom) shoes were evaluated in this study. The Heel-Toe Drop is the difference in sole height between the toe and the heel of a shoe. Rigidity is defined as the amount of bend in the shoe in the sagittal plane when bent by a study investigator. Rigidity is rated as either minimal (shoe bends >45°), moderate (shoe bends <45°), or rigid (shoe bends <10°).^1^ The minimalist shoe had a minimalist index score of 25/25, where a higher score means a higher degree of minimalism.^12^ The ASICS Kayano had a minimalist index score^12^ of 2/25.

Two studies have investigated the effect of minimalist shoes on patellofemoral joint forces during running in adolescents with patellofemoral pain. One compared minimalist shoes (minimalist index of 25/25, where a higher score indicates a higher degree of minimalism)^12^ to traditional school shoes (minimalist index score of 2/25),^28^ and the other with neutral shoes (ie, athletic shoes with no motion-control properties; minimalist index score of 9/25).^29^ Both studies observed a reduction in 2-dimensional (2D) (sagittal plane only) patellofemoral joint forces when participants ran on a treadmill in minimalist shoes. The reduction in patellofemoral force in the minimalist shoe is likely due to decreased peak knee flexion and quadriceps muscle force,^31^ accompanied by an increase in gastrocnemius force that attenuates greater ankle force.^39^ However, neither of these studies compared the minimalist shoes with motion control shoes, which adolescents commonly wear during running. Additionally, using 2D patellofemoral joint models to estimate patellofemoral joint contact forces during treadmill running may not accurately reflect the 3D patellofemoral forces experienced during everyday activities, such as overground running.^42,55^ As such, studies that utilize 3D patellofemoral joint models to estimate the effects of footwear on patellofemoral joint forces during overground running are warranted.

Accordingly, the primary aim of this study was to use electromyography (EMG)-informed neuromusculoskeletal modeling to compare patellofemoral joint and muscle forces during overground running in adolescents with patellofemoral pain while wearing minimalist and motion-control shoes. We hypothesized that patellofemoral joint, patellar, and quadriceps forces would be lower, whereas gastrocnemius forces would be higher when adolescents ran in minimalist shoes compared with motion-control shoes.

## Methods

This within-session randomized crossover study is reported according to the CONSORT (Consolidated Standards of Reporting Trials) 2010 statement: extension to randomized crossover trials^8^ and Report-PFP checklist^2^ (Supplementary Table 5, available in the online version of this article). A crossover design was chosen because the effect of footwear on patellofemoral joint force is immediate and reversible, allowing each participant to serve as their own control. Study procedures were approved by the institution's Human Research Ethics Committee (reference No. 25516). All participants were informed of the study aims, the testing procedures (including the use of minimalist and motion-control shoes during running), and the anticipated results. All participants provided written informed consent electronically.

### Participants

#### Sample Size

An a priori sample size calculation was conducted to detect a minimum between-group difference of 4.2 N/Kg (in our sample, where mean weight was 66.7 kg, this corresponds to a difference of 280.1 N) using an effect size of 0.3, and an assumed pooled standard deviation of 14 N/Kg (with our sample, this corresponds to a pooled standard deviation of 933.8 N).^3^ With 80% power, an assumed within-participant intraclass correlation^44^ of 0.722, and a significance level of .05, we determined that a total sample size of 51 was required.

#### Recruitment

Participants were recruited from the greater Melbourne area via social media, local schools, referrals from allied health professionals, and flyers on noticeboards around the University of Melbourne. Recruitment took place from March 2023 to September 2024. Interested individuals completed an online screening survey in REDCap (Research Electronic Data Capture)^19,20^ to determine their potential eligibility. Individuals deemed eligible were provided with information about the study, including the plain-language statement, and eligibility was confirmed through a final telephone screening.

#### Inclusion/Exclusion Criteria

All participants had to be aged 12 to 19 years at the time of testing. Established clinical guidelines informed our definition of patellofemoral pain.^5,57^ Participants were included if they (1) had pain at the front of the knee that was aggravated by running, squatting, kneeling, or descending stairs; (2) had knee pain in the previous week ≥3 on an 11-point numerical rating scale (NRS), where 0 indicates no pain and 10 worst pain imaginable; and (3) knee pain was present for >2 months. Participants were excluded if they (1) had pain that commenced after a traumatic injury; (2) had previous knee surgery; (3) had been diagnosed in the previous 12 months by a health professional as having a knee condition other than patellofemoral pain, or had evidence of any other knee injury (eg, ligament or meniscal injury as assessed by an osteopath with 5 years’ experience); (4) had other muscle or joint pain anywhere in the body that was worse than their patellofemoral pain; (5) had any systemic inflammatory joint disease; (6) had any neurological condition affecting the spine or either lower limb; (7) could not fit into a female or male shoe size 6-11 or 6-13 US, respectively (due to restriction in available study shoe sizes); (8) were not physically active (ie, not currently participating in planned sports and exercise activities); (9) did not understand written and spoken English; or (10) had a body mass index (BMI) of >35 kg/m^2^ due to difficulties with data collection and movement artefact.

### Descriptive Measures

Demographic data were collected, including sex at birth, height, body mass, and BMI. Physical activity was assessed using the Physical Activity Questionnaire for Adolescents (PAQ-A). We reported the total PAQ-A score and the specific physical activities participants had engaged in over the previous 7 days.^22^ The worst pain experienced in the previous week was rated using an 11-point NRS, where 0 indicated no pain at all, and 10 represented the worst pain imaginable. Participants also completed the Knee injury and Osteoarthritis Outcome Score for children (KOOS-Child). The KOOS-Child comprises 5 subscales: symptoms, pain, activities of daily living, sport and play, and quality of life.^32^ Fear of movement was quantified using the Tampa Scale for Kinesiophobia.^13^

### Procedures

This study was conducted within the institution's biomechanical laboratory. If participants had bilateral symptoms, the most symptomatic knee was used. If both were equally symptomatic, the knee with the longest symptom duration was used. For the first 23 participants, muscle activity was recorded using a wireless telemetered 16-channel Telemyo DTS system (Noraxon) sampling at 1200 Hz. For the remaining 28 participants, an 18-channel EMG system (PicoX Cometa) sampling at 2000 Hz was used. The change to the EMG system was necessary because the original system became obsolete.

Following the Surface Electromyography for the Non-Invasive Assessment of Muscle guidelines, EMG data were collected from 8 muscles of the lower limb: rectus femoris, vastus lateralis, vastus medialis, biceps femoris, semitendinosus, tibialis anterior, and the lateral and medial gastrocnemius. Electrode attachment sites were identified by 1 investigator (S.K.) and marked with a nonpermanent marker. If required, hair was shaved from the attachment sites. The skin was prepared using light skin abrasion and alcohol, and EMG surface electrodes were placed on the muscles of interest.

All participants performed 2 maximum voluntary isometric contractions (MVICs) to elicit maximal EMG amplitudes for each instrumented muscle by performing knee flexion and extension, as well as ankle plantar- and dorsi-flexion, using a HUMAC Norm Isokinetic Dynamometer (CSMi). For each instrumented muscle, the peak amplitude was obtained from either the MVIC or the running task and used to normalize EMG during the running task. The MVIC test protocol was adapted from previous studies.^6,14,52^ The MVIC testing setup is available in the Supplementary File (available online).

Kinematic data were recorded using a 12-camera Vicon motion analysis system (Vicon) at 200 Hz, which tracked the spatial trajectories of 40 reflective markers placed on bony landmarks of the torso, lower limbs, and shoes. Our complete marker set is available in Supplementary Table 4 (available online). Ground reaction forces were recorded using an embedded force plate sampling at 2400 Hz (AMTI Inc). A static trial was recorded before the running trials to scale the musculoskeletal model anthropometry.

### Shoes and Randomization

We defined minimalist and motion-control shoes using published criteria.^35^ We then chose 2 commercially available shoes that adolescents preferred:^36^ the Vivo Primus Lite II for the minimalist shoe and an ASCIS Kayano 25 for the motion control shoe (Figure 1). The sequence of shoe testing conditions (minimalist or motion control first) was assigned in random, permuted blocks of 2 and 4, with a 1-to-1 allocation ratio, to mitigate the risk of period effects. A statistician, who had no involvement in the data collection process, prepared the randomization schedule. It was shared with another researcher, who then revealed and delivered the sequence to the primary researcher, who randomized each participant to their assigned sequence.

### Running Task

Participants were given time to familiarize themselves with running in the laboratory, during which they ran up to 5 minutes in each pair of shoes. Participants ran along a 15-m walkway with an embedded force plate. Participants ran at a self-selected speed and were given standardized instructions to “Run as normally as possible and do not overthink your running style. Run as if you are going for a run on the street.” Participants were also prompted to run faster than a jog but slower than a sprint. This prompt was given as large variations in running speed can alter lower limb biomechanics.^56^ Three successful running trials were recorded for each shoe condition. Trials were considered successful if (1) participants ran at the same speed ±10% both within and between the 2 shoe conditions; and (2) the foot of the most symptomatic limb made complete contact with the concealed force plate (ie, the foot was inside the borders of the force plate). Running speed was measured using SmartSpeed Plus timing gates (VALD Performance).

### Computational Modeling and Data Analysis

Kinematic and ground reaction force data were low-pass filtered using a 4th-order zero-lag Butterworth filter with cutoff frequencies of 6 and 10 Hz, respectively. EMG signals were high-pass filtered, with a 20 Hz cut-off frequency. To create linear envelopes, the filtered EMG signals were full-wave rectified and then low-pass filtered with a 2nd-order zero-lag Butterworth filter at 6 Hz. Foot strike and toe off events were identified from the forceplate data using a 60 N threshold of vertical ground-reaction force.

The patellofemoral joint contact force and muscle forces during the running tasks were estimated using a customized OpenSim-MATLAB 2024b (Mathworks Inc) interface and the Calibrated EMG-informed Neuromusculoskeletal Modeling toolbox (CEINMS).^37^ A generic full-body musculoskeletal model was used in OpenSim (Version 3.3).^40^ The model had 3 (flexion/extension, adduction/abduction, and internal/external rotation), 1° (flexion/extension), and 2° (plantarflexion/dorsiflexion, and inversion/eversion) of freedom at the hip, knee, and ankle, respectively. The hip joint center was derived from a regression equation using the pelvic markers.^15^ Model segments and mass inertial properties were linearly scaled to match each subject's anthropometry using distances between marker pairs acquired during the static pose trial. After linear scaling, Hill-type muscle parameters (eg, optimal muscle fiber and tendon slack lengths) were optimized using an anthropometric algorithm.^30^ From each running trial, joint kinematics and kinetics, as well as muscle moment arms, were determined using inverse kinematics, inverse dynamics, and OpenSim's muscle analysis tools, respectively.^45^

**Figure 2.**
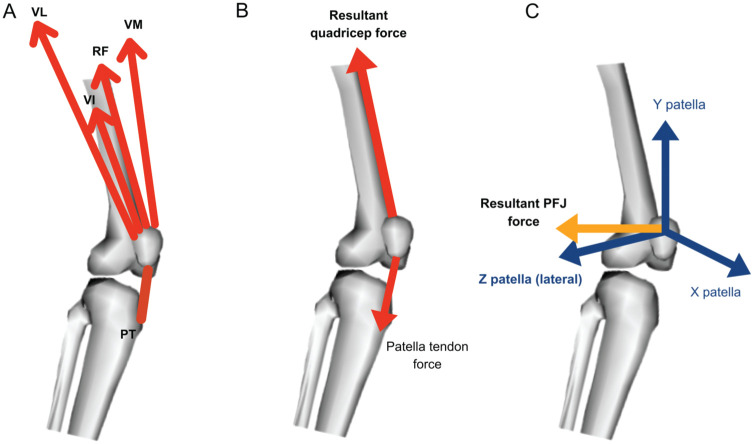
Simplified diagram of the 3-dimensional patellofemoral joint (PFJ) model.^50^ (A) The vastus lateralis (VL), vastus intermedius (VI), rectus femoris (RF), vastus medialis (VM), and patella tendon (PT) vectors were calculated from each participant's scaled model and experimental measurements. (B) Next, the patellar tendon force was obtained based on the assumption of zero Y-axis friction in the patellofemoral joint. (C) From this, the patella forces in the X, Y, and Z directions were calculated. These were summed to calculate the resultant patellofemoral joint force.

Then, the acquired joint kinematics and kinetics, along with measured muscle moment arms and activations (ie, EMG signals from the measured muscles), were used to calibrate a neuromusculoskeletal model using Calibrated EMG-Informed Neuromusculoskeletal Modeling (CEINMS).^37^ After calibration, CEINMS was used in EMG-assisted mode to estimate the forces in 8 lower limb muscles mapped to 11 musculotendon units.^43^ The remaining 33 lower limb musculoskeletal units were synthesized using an optimization criterion. The objective of the EMG-assisted mode was to minimize tracking errors in joint moments and muscle activity (ie, experimental measures vs modeling predictions). We quantified the correlation between inverse-dynamics-derived joint moments and their corresponding model predictions using the Pearson correlation coefficient (*r*) and the root-mean-square error (Supplementary File, available online). The resultant patellofemoral joint contact force was estimated using a previously published algorithm that computes the static equilibrium of 3D muscle forces acting on the patellar center of mass (Figure 2).^50^

### Outcomes

The stance-phase data for each participant's most symptomatic limb were averaged across 3 successful trials (steps). All biomechanical variables for each stance phase of running were spline-interpolated to 101 time points. The primary outcome was the peak resultant patellofemoral joint force (N). Secondary outcomes included peak lateral patellar force (defined as the peak force in the Z direction acting on the patellar [Figure 2C] or frontal plane, N); peak quadriceps (sum of peak vastus lateralis, vastus medialis, vastus intermedius, and rectus femoris forces) and gastrocnemius (sum of peak medial and lateral gastrocnemius forces) muscle forces (N); peak knee extension, hip extension, and ankle plantar flexion moments (N·m); and peak knee flexion angle (deg). Immediately after the running task, participants rated the comfort of each shoe and the pain in their experimental knee using 11-point numerical rating scales, with terminal descriptors of zero (extremely uncomfortable) to 10 (extremely comfortable) and zero (no pain at all) to 10 (the worst pain they had ever felt). For each outcome, we additionally calculated the mean and standard deviation. For each biomechanical outcome, we calculated the mean difference and standard deviation and the mean percentage difference using the motion control mean as the denominator.

### Statistical Analyses

#### Peak

Our statistical analyses were conducted using Stata (StataCorp). Peak outcomes were compared using paired *t* tests with an alpha level set to .05. We also conducted a sensitivity analysis to assess whether the EMG system influenced our primary outcome by running a linear regression analysis of our primary outcome with and without the EMG system as a covariate.

#### Waveform

The effects of footwear may extend beyond predefined regions (ie, peaks). Thus, we analyzed the effects of the 2 footwear conditions on resultant patellofemoral joint force, lateral patellar force, quadriceps muscle forces, and gastrocnemius muscle forces across their respective waveforms, using 1D statistical parametric mapping with paired *t* tests.^33^ This was conducted within MATLAB using the package SPM1D (spm1d v0.4, http://www.spm1d.org). Statistical Parametric Mapping (SPM) works well for biomechanical waveforms as they are smooth, sampled above the Nyquist frequency, and bounded by time.^33,34^ The critical threshold value (t*) and the SPMt were calculated^34^ in MATLAB, with an alpha level of .05.

## Results

Demographic data are presented in Table 1. The participant characteristics were similar across the 2 sequences (ie, participants randomized to wear minimalist shoes first or motion-control shoes first). The mean self-selected running speed in both shoes was 3.8 ± 0.4 m/s. On average, the motion-control shoes were rated more comfortable than the minimalist shoes (minimalist, 2.5 ± 1.8 points; motion-control, 6.9 ± 1.4 points; *P*≤ .01). The mean pain immediately after running in each shoe condition was similar (minimalist, 3.6 ± 2.2 points; motion-control, 2.9 ± 1.9 points; *P* = .11). The score on PAQ-A indicated adolescents performed a moderate amount of physical activity over the past 7 days, with the most frequent sports being jogging or running (n = 43/51 [84%]), walking for exercise (n = 42/51 [82%]), basketball (n = 23/51 [45%]), and bicycling (n = 18/51 [35%]) (Supplementary Table 2, available online).

**Table 1:** Participant Characteristics by Sequence and Total*^*a*^*

Patient Characteristics	Sequence 1:*^*b*^* Minimalist to Motion-Control n = 24	Sequence 2:*^*b*^* Motion Control to Minimalist n = 27	Total N = 51
Female sex	11 (46)	12 (44)	23 (45)
Age, years	16.9 (1.8)	16.8 (2.1)	16.9 (2)
Height, cm	167.8 (9.7)	173.0 (10.8)	170.5 (10.5)
BM, kg	65.9 (15)	67.4 (13.4)	66.7 (14.1)
BMI, kg/m^2^	23.2 (4)	22.5 (3.8)	22.7 (3.8)
Worst pain in the previous week	5 (1.4)	5.2 (1.6)	5.1 (1.5)
Most painful knee, right	13	10	23
Duration of symptoms, months	13 (5.8-30)	20 (8.5-38)	18 (6.7- 36)
KOOS-Child			
Symptoms	78.6 (12.2)	79.6 (10.5)	79.1 (11.2)
Pain	69.1 (13.2)	69.2 (12.6)	69.2 (12.7)
Activities of daily living	82.2 (12.6)	83.4 (11)	82.8 (11.7)
Sport and play	62.5 (15.8)	65.3 (13.1)	64 (14.3)
Quality of life	64.8 (15.2)	62.3 (13.5)	63.5 (14.2)
Tampa	34.6 (6)	36.4 (4.8)	35.6 (5.4)
PAQ-A	2.3 (0.7)	2.4 (0.7)	2.4 (0.7)

a Data are presented as means (SD), n (%), or median (IQR). BM, body mass; BMI, body mass index; IQR, interquartile range; KOOS, Knee injury and Osteoarthritis Outcome Score; NRS, numerical rating scale; PAQ-A, Physical Activity Questionnaire for Adolescents; Tampa, Tampa Scale for Kinesiophobia.

Scales: Worst pain in the previous week rated on an NRS (scored from 0 to 10; higher = greater pain). KOOS-Child subscales (scored from 0 to 100; higher = milder symptoms). PAQ-A (scored from 0 to 5; higher = more physical activity in the previous week). The Tampa Scale for Kinesiophobia (scored from 17 to 68; higher = more fear of movement).

b The sequence is the randomized order of shoe testing (minimalist or motion control first).

### Primary Outcome

Compared with the motion-control shoes, running in the minimalist shoes reduced peak resultant patellofemoral joint force by 7.5% (mean difference [MD], −363.2 N [95% CI, −666.8 to −59.5]; *P* = .02) (Table 2).

**Table 2:** Comparison of Peak Variables Between the Minimalist and Motion-Control Shoes*^*a*^*

Variable, N	Minimalist, Mean (SD)	Motion-Control Mean, (SD)	MD (95% CI)	Mean % Difference	*P*
Resultant PFJ	4478.6 (1805.4)	4841.7 (1869.3)	−363.2 (−666.8 to −59.5)	7.5	.02
Lateral patella	2390.3 (2125)	2593 (2258.9)	−202.7 (−379 to −26.4)	7.8	.02
Quadriceps	6943.7 (2003)	7148.8 (1922.1)	−205.1 (−515.7 to 105.5)	2.9	.12
Gastrocnemius	2136.0 (622)	1686.7 (668.7)	+449.3 (+298.6 to +560)	26.6	<.01

a MD, mean difference; PFJ, patellofemoral joint.

### Secondary Outcomes

Compared with the motion-control shoes, running in the minimalist shoes led to a reduction of 7.8% in peak lateral patellar force (MD, −202.7 N [95% CI, −379 to −26.4]) and a 26.6% increase in peak gastrocnemius muscle forces (MD, 449.3 N [95% CI, 298.6 to 560]). In our sample, running in the minimalist shoe did not meaningfully alter quadriceps muscle forces compared with the motion control shoe (Table 2). Compared with the motion-control shoe, running in the minimalist shoe led to a reduction in the peak hip extension moment (MD, −8.5 N·m [95% CI, −16.2 to −0.8]), peak knee extension moment (MD, −14.1 N·m [95% CI, −21.0 to −7.2]), peak knee flexion angle (MD, −1.5° [95% CI, −2.3 to −0.6]), and an increase in the ankle plantar flexion moment (MD, 12.1 N·m [95% CI, 7.4 to 16.8]) (Supplementary Table 1, available online).

### Statistical Parametric Mapping

**Figure 3.**
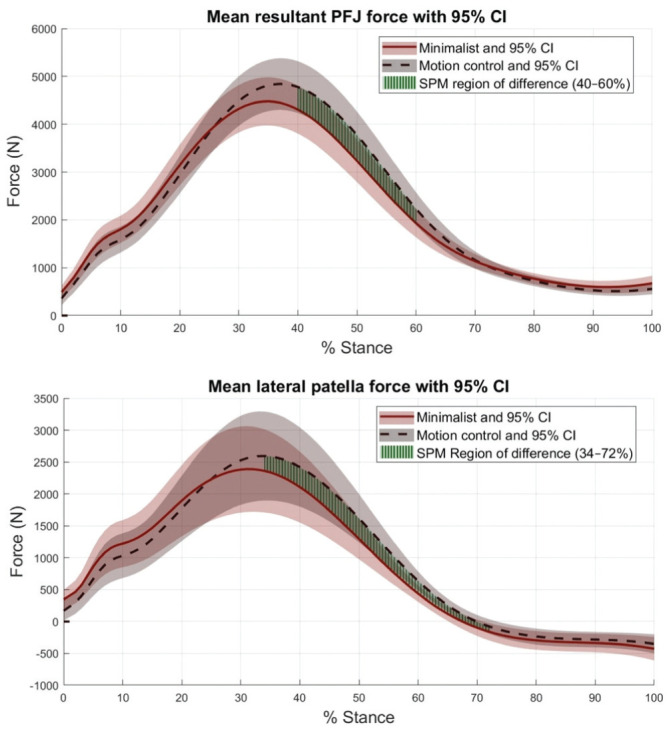
Mean resultant patellofemoral joint (PFJ) force (top) and lateral patellar force (bottom), 95% CIs (shaded) and statistical parametric mapping (SPM) using paired *t* tests across the stance phase of running while wearing minimalist shoes (red solid line) and motion-control shoes (black dashes). The green region represents a difference between the waveforms in the SPM analyses.

**Figure 4.**
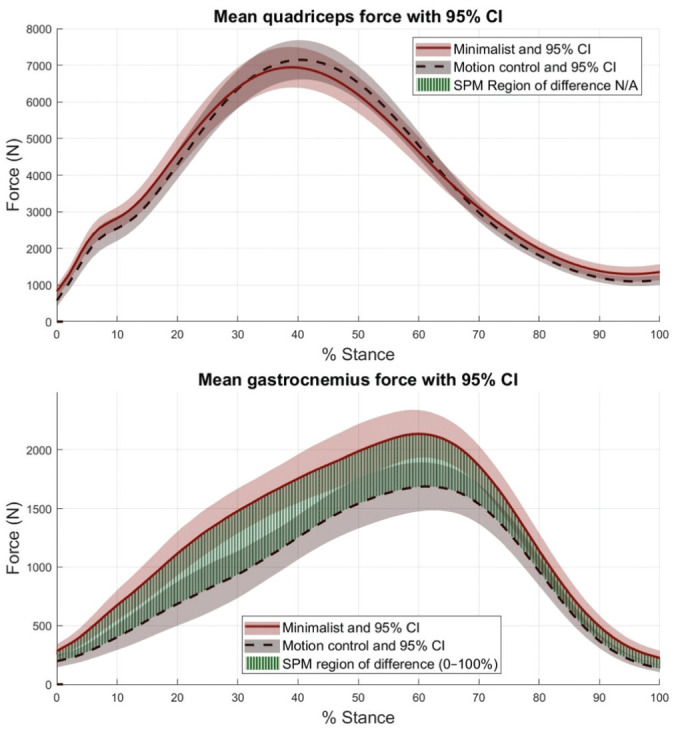
Mean quadriceps (top) and gastrocnemius (bottom) muscle force, 95% CIs (shaded), and Statistical Parametric Mapping (SPM) using paired *t* tests across the stance phase of running while wearing minimalist shoes (red solid line) and motion-control shoes (black dashes). The green region represents a difference between the waveforms in the SPM analyses.

According to the SPM analyses, running in the minimalist shoes reduced the resultant patellofemoral joint force during 40% to 60% of stance compared with the motion-control shoes, the lateral patellar force during 34% to 72% of stance (Figure 3), and increased the gastrocnemius muscle force during 0% to 100% of stance (Figure 3). The quadriceps waveforms were similar across the entire stance phase (Figure 4). The MDs across the waveforms and SPMt graphs are provided in the supplemental file (available online).

### Sensitivity Analysis

Results from our linear regression showed that the association between the minimalist and motion-control peak resultant patellofemoral joint force did not meaningfully change with the inclusion of the EMG system as a covariate. For every 1 N of force while running in the motion control shoe, there was 0.8 N (95% CI, 0.6-1) in the minimalist shoe without the EMG covariate and 0.8 N (95% CI, 0.6-0.9) in the minimalist shoe with the EMG covariate, indicating that the change in EMG system did not influence the primary outcome (Supplementary Table 3, available online).

## Discussion

### Summary of Findings

**Figure 5.**
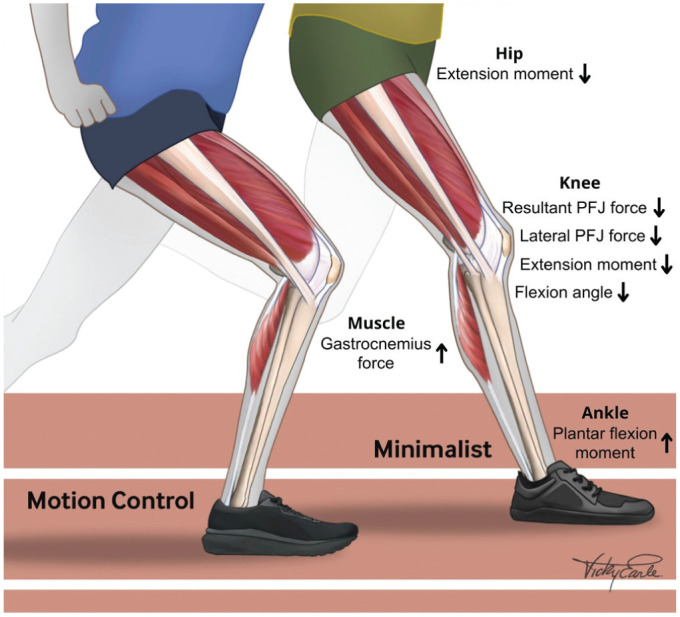
Summary of effects while running in a minimalist shoe compared with a motion-control shoe.

Using EMG-informed neuromusculoskeletal modeling, the primary aim of this study was to compare patellofemoral joint and muscle forces during overground running in adolescents with patellofemoral pain while wearing minimalist and motion-control shoes. As hypothesized, compared with the motion control shoe, running in the minimalist shoe reduced peak resultant patellofemoral joint and lateral patellar forces while increasing gastrocnemius forces. However, contrary to our hypothesis, the minimalist shoe did not significantly alter quadriceps muscle forces compared with the motion-control shoe. We have summarized the effects of minimalist shoes compared with those of motion-control shoes in Figure 5.

### Effects of Minimalist Shoes on the Resultant Patellofemoral Joint Force

The reduction in the resultant patellofemoral joint force when running in minimalist shoes is partly driven by decreased knee flexion, which reduces the internal knee extension moment and quadriceps demand, thereby lowering the resultant patellofemoral joint force.^46^ Indeed, knee flexion and the knee extension moment were found to be reduced in the minimalist shoe, and, although the quadriceps muscle forces were not different across shoe conditions based on statistical significance, a difference may have existed with a larger sample size, which may have narrowed the confidence intervals. However, the MD in quadriceps forces between the shoes was only 2.9%, which is unlikely to be clinically meaningful. An increase in cadence may have contributed to the reduction in knee flexion. Although cadence was not directly measured, previous studies have shown that minimalist shoes are associated with shorter step length and increased cadence,^4,47,49^ which are known to decrease patellofemoral joint force.^3,26^ In addition, characteristic features of minimalist shoes—including the combination of reduced heel height,^47^ decreased weight,^11^ lower heel-to-toe drop,^58^ and enhanced flexibility^11^—may have also contributed to a redistribution of mechanical work (ie, force production and attenuation) from the knee to the ankle and the gastrocnemius,^39,21^ thereby reducing patellofemoral joint force.^46^ Finally, it is possible that other unmeasured muscles, such as the soleus or those that span the hip joint, may have contributed to the reduction in the knee flexion angle and/or patellofemoral joint force.^25,38^

### Effects of Minimalist Shoes on Lateral Patellar Force

On average, participants in our study ran with a lateral rather than a medial patellar force in both shoe conditions. Despite the proposed mechanism of motion-control shoes being a reduction in foot pronation, tibial and femoral internal rotation, and lateral force on the patella,^23,54^ the peak lateral force was not reduced compared with minimalist shoes, which have no motion-control properties. Instead, the lateral force was higher. This may indicate that any reduction in foot pronation (and consequently tibial or femoral rotation) produced by motion-control shoes was insufficient to reduce lateral patellar force in the context of the concomitant increase in the knee flexion angle (and thus resultant patellofemoral joint force) when running in these shoes.

### Statistical Parametric Mapping

In addition to our discrete comparisons, we also analyzed the entire waveform across stance using SPM. Compared with the motion control shoe, SPM of the continuous waveforms showed that changes from wearing the minimalist shoe extended beyond their peaks into the second half of the stance. This suggests a sustained reduction in patellofemoral joint forces, with important clinical implications. Patellofemoral pain is particularly prevalent among active adolescents, likely due to sustained exposure to high forces across multiple loading cycles (ie, cumulative force).^48^ The sustained reduction in patellofemoral joint force from wearing a minimalist shoe (relative to a motion control shoe) could be replicated across multiple loading cycles, resulting in exponential reductions in joint force. As such, clinicians aiming to reduce cumulative patellofemoral joint force may recommend minimalist shoes for adolescents with patellofemoral pain.

Our findings align with previous research, which demonstrated a reduction in patellofemoral joint reaction force during the loading phase of running in minimalist shoes compared with traditional school^28^ and neutral shoes.^29^ However, these studies estimated patellofemoral joint force in the sagittal plane during treadmill running, thereby overlooking potential changes in the transverse and frontal planes. The novel finding from our study is that the resultant patellofemoral joint force and lateral patellar force are reduced when adolescents with patellofemoral pain run overground in minimalist shoes compared with motion-control shoes. In contrast, previous research has shown that minimalist shoes increase medial tibiofemoral contact forces compared with stable supportive shoes during walking in people with medial knee osteoarthritis.^51^ As such, minimalist shoes should be used selectively to reduce patellofemoral joint force only, rather than overall knee joint force.

### Clinical Implications

It is unclear whether the biomechanical changes observed in our study will lead to symptomatic improvement over time in adolescent patellofemoral pain. The pathophysiology of patellofemoral pain involves high, sustained forces that result in tissue (eg, subchondral bone) overload within the patellofemoral joint,^9^ particularly on the lateral patellar facet. Although the resultant and lateral patellar forces were lower in the minimalist shoe than in the motion-control shoe, this was not accompanied by lower pain. Consequently, even though minimalist shoes reduced cross-sectional patellofemoral joint forces compared with motion-control shoes, it is unknown whether this reduction in forces leads to a reduction in tissue overload and, thus, pain over time. Further longitudinal research is needed to determine whether a cumulative reduction in force resulting from long-term use of a minimalist shoe leads to reduced pain. However, it is also possible that an adaptation to minimalist shoes may occur after prolonged use. Habituation to barefoot running has been shown to increase mean vertical ground reaction force and shift the foot strike toward the forefoot over time compared with acute running.^18^ Both biomechanical changes may alter patellofemoral joint forces and, theoretically, pain. Consequently, clinicians should also consider these potential habituation effects if recommending minimalist footwear, recognizing that short-term responses may not reflect long-term adaptations. Finally, on average, adolescents in our study rated the minimalist shoe as less comfortable than the motion-control shoe. As such, reduced comfort with minimalist shoes may limit their uptake among adolescents with patellofemoral pain, even if they are found to be clinically effective.

### Strengths and Limitations

The strengths of our study include the use of an EMG-informed 3D neuromusculoskeletal patellofemoral joint model, which provides a more robust, physiologically accurate measure of in vivo force than traditional 2D patellofemoral joint models.^20^ The sample was adequately powered and represents the largest 3D patellofemoral EMG-informed modeling study conducted to date in adolescents. We also used overground running to assess joint forces, which more accurately reflect adolescents’ everyday movements than treadmill running.^42,55^ There were also some limitations to consider. Our patellofemoral joint model did not incorporate patient-specific factors—such as morphology, cartilage thickness, and soft tissue anatomy. The relationship between joint forces and tissue loading is likely nonlinear due to the variations in these patient-specific factors. Consequently, future research integrating magnetic resonance imaging data is essential for understanding the internal loading environment of the patellofemoral joint. We also did not include adolescents without patellofemoral pain, who may possess different knee morphologies that lead to different biomechanical responses to footwear. However, a recent systematic review reported reduced patellofemoral joint force during running with minimalist shoes compared with “conventional” shoes in adults with and without patellofemoral pain.^19^ We did not assess pubertal maturation or hormonal status; as such, it is unknown whether these factors influence the effect of shoes on patellofemoral joint force. We measured cross-sectional joint force during a single instance of running in a laboratory setting. This may not mimic all real-world loading activities that adolescents undertake (eg, walking or sport-specific movements), nor does it allow us to determine whether differences between footwear types are maintained if they are worn for an extended period of time. Given that all testing took place within a single session, we were unable to implement an extended washout period. To mitigate any period effects, the order in which the participants wore the shoes was randomized. Nonetheless, in future studies, potential carryover effects could be mitigated by extending the washout period. Finally, we utilized 2 different EMG systems to measure muscle activity, which may have influenced our primary outcome. However, the negligible difference in our sensitivity analysis indicated that changes in the EMG system did not affect our primary outcome.

## Conclusion

As hypothesized, running in minimalist shoes reduced peak resultant patellofemoral joint and lateral patellar forces compared with running in motion-control shoes, while gastrocnemius forces were increased. Running in minimalist shoes did not meaningfully alter quadriceps muscle forces compared with motion-control shoes.

## Supplemental Material

sj-docx-1-ajs-10.1177_03635465261443316 – Supplemental material for The Effect of Minimalist Versus Motion Control Shoes on Patellofemoral Joint Forces in Adolescents With Patellofemoral Pain During Running: A Randomized Crossover Study Supplemental material, sj-docx-1-ajs-10.1177_03635465261443316 for The Effect of Minimalist Versus Motion Control Shoes on Patellofemoral Joint Forces in Adolescents With Patellofemoral Pain During Running: A Randomized Crossover Study by Samual A. Kayll, Rana S. Hinman, Adam L. Bryant, Kim L. Bennell, Patrick L. Rowe, Pei Wei Chi, Scott Starkey, Fiona McManus, David Saxby and Kade L. Paterson in The American Journal of Sports Medicine
