# Supplementary material for: The Effect of Minimalist Versus Motion Control Shoes on Patellofemoral Joint Forces in Adolescents With Patellofemoral Pain During Running: A Randomized Crossover Study
Source: Am J Sports Med. 2026 May 16;54(8):1933–43. doi: 10.1177/03635465261443316 (PMC13279980; doi:10.1177/03635465261443316)
Supplement: sj-docx-1-ajs-10.1177_03635465261443316 – Supplemental material for The Effect of Minimalist Versus Motion Control Shoes on Patellofemoral Joint Forces in Adolescents With Patellofemoral Pain During Running: A Randomized Crossover Study [file sj-docx-1-ajs-10.1177_03635465261443316.docx]

| ***Moments (Nm)*** | Minimalist | Motion Control | MD | 95% CI | P Value |
| --- | --- | --- | --- | --- | --- |
| Knee Extension | 174.7 (42.8) | 188.8 (45.0) | - 14.1 | -21.0 to -7.2 | <0.01 |
| Ankle Plantar Flexion | 193.6 (46.6) | 181.5 (44.5) | 12.1 | 7.4 to 16.8 | <0.01 |
| Hip Extension | 116.7 (41.9) | 125.3 (43.6) | - 8.5 | -16.2 to - 0.8 | 0.03 |
| ***Kinematics*** | Minimalist | Motion Control | MD | 95% CI | P Value |
| Knee Flexion | 45.6 (6.3) | 47.1 (6.6) | - 1.5 | - 2.3 to - 0.6 | <0.01 |

Supplementary table 1: Joint moments and knee flexion

Supplementary table 2: The physical activities that the adolescents performed over the previous seven days according to the Physical Activity Questionnaire for Adolescents (PAQ-A).

| **Physical activity** | **# (%)^a^** |
| --- | --- |
| Jogging or running | 43 (84) |
| Walking | 42 (82) |
| Basketball | 23 (45) |
| Bicycling | 18 (35) |
| Other | 16 (31) |
| Skipping | 12 (24) |
| Dance | 10 (20) |
| Tag | 10 (20) |
| Soccer | 8 (16) |
| Swimming | 7 (14) |
| Football | 6 (12) |
| Aerobics | 6 (12) |
| Volleyball | 5 (10) |
| Rowing/Canoeing | 4 (8) |
| Ice Skating | 2 (4) |
| Ice Hockey | 1 (2) |
| Floor Hockey | 1 (2) |
| Badminton | 1 (2) |

^a^The percentage of participants (n = 51) who selected each activity**
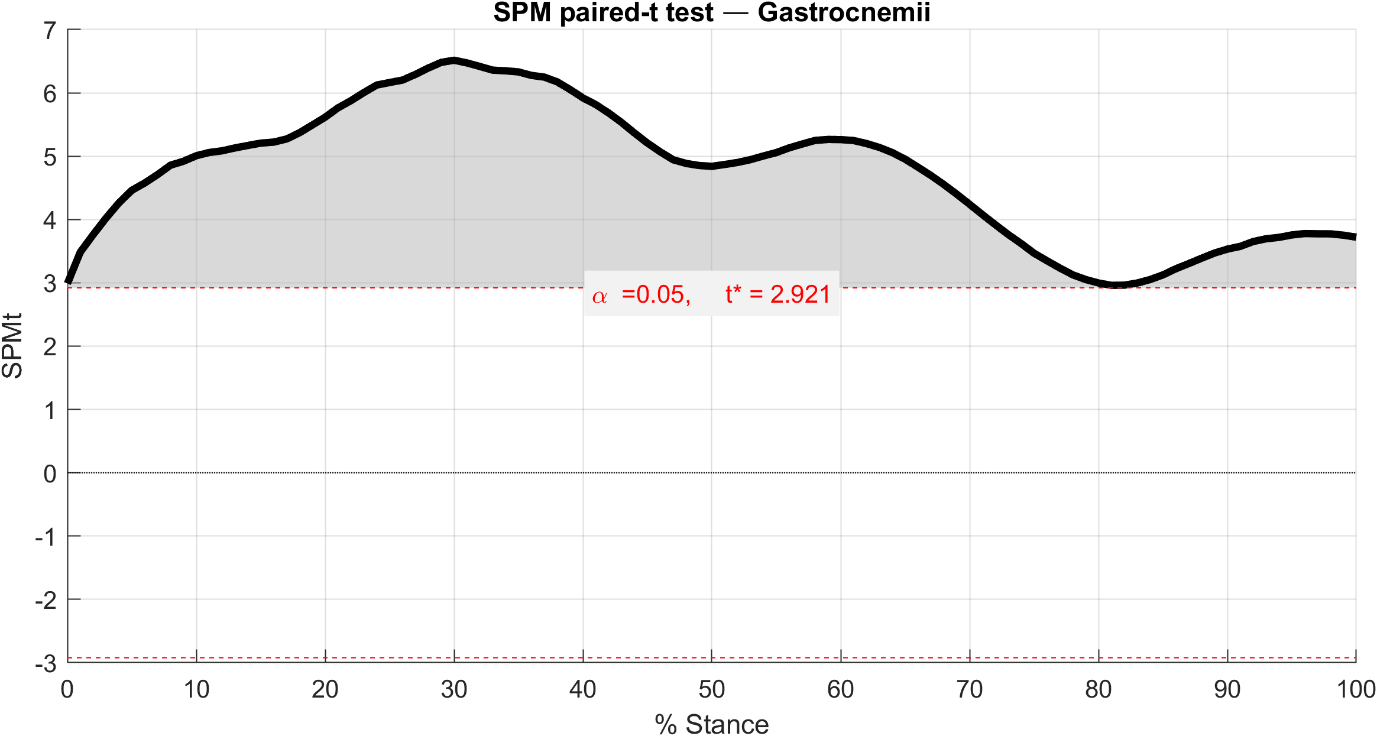
**

**
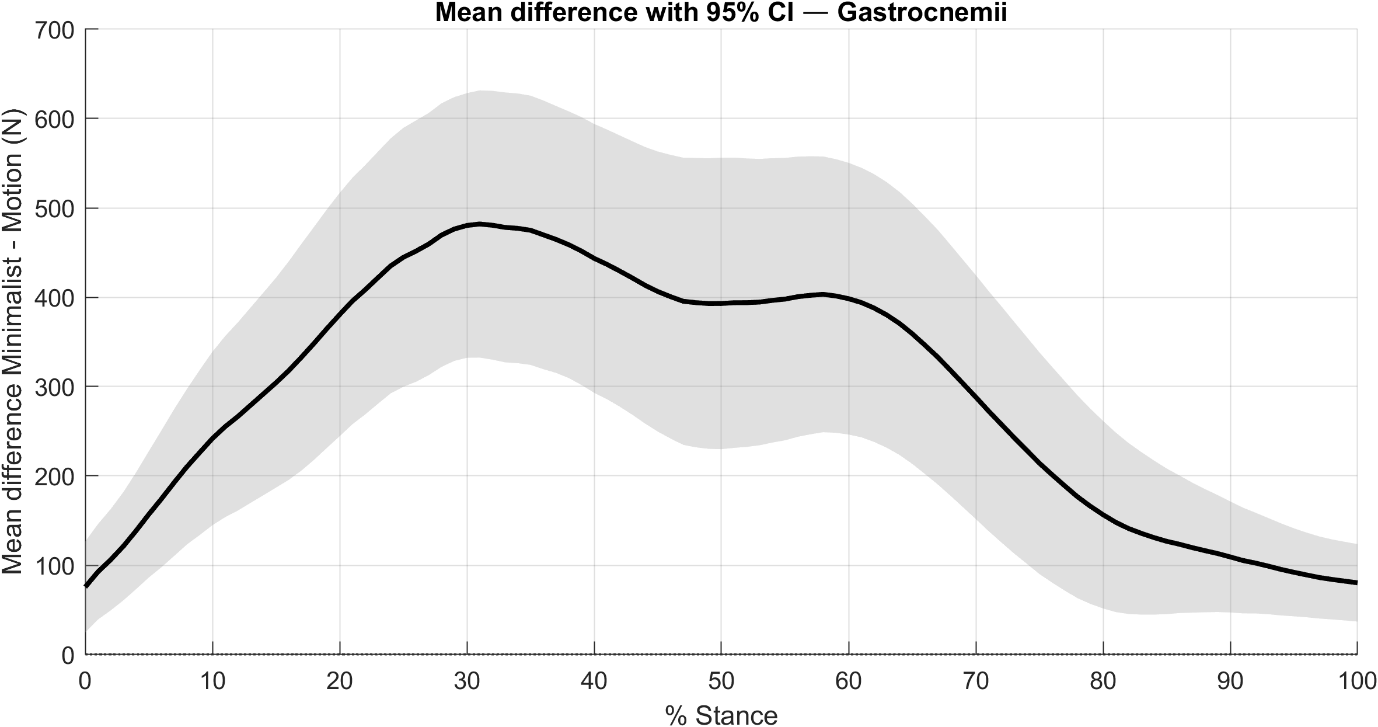
**

Gatrocnemius t graph (top) and plotted mean difference with 95% confidence interval (CI) (bottom). The mean difference is represented as the mean minimalist force minus the mean motion force.

**
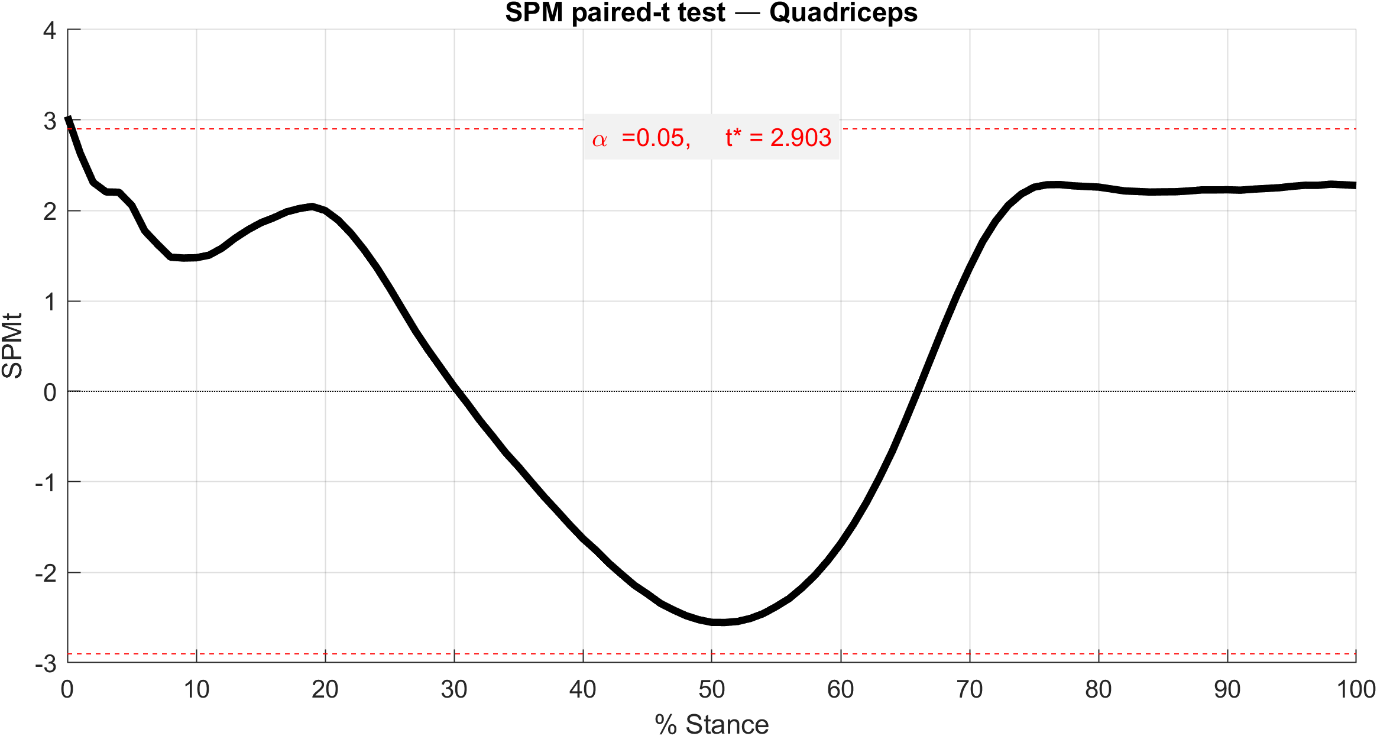
**

**
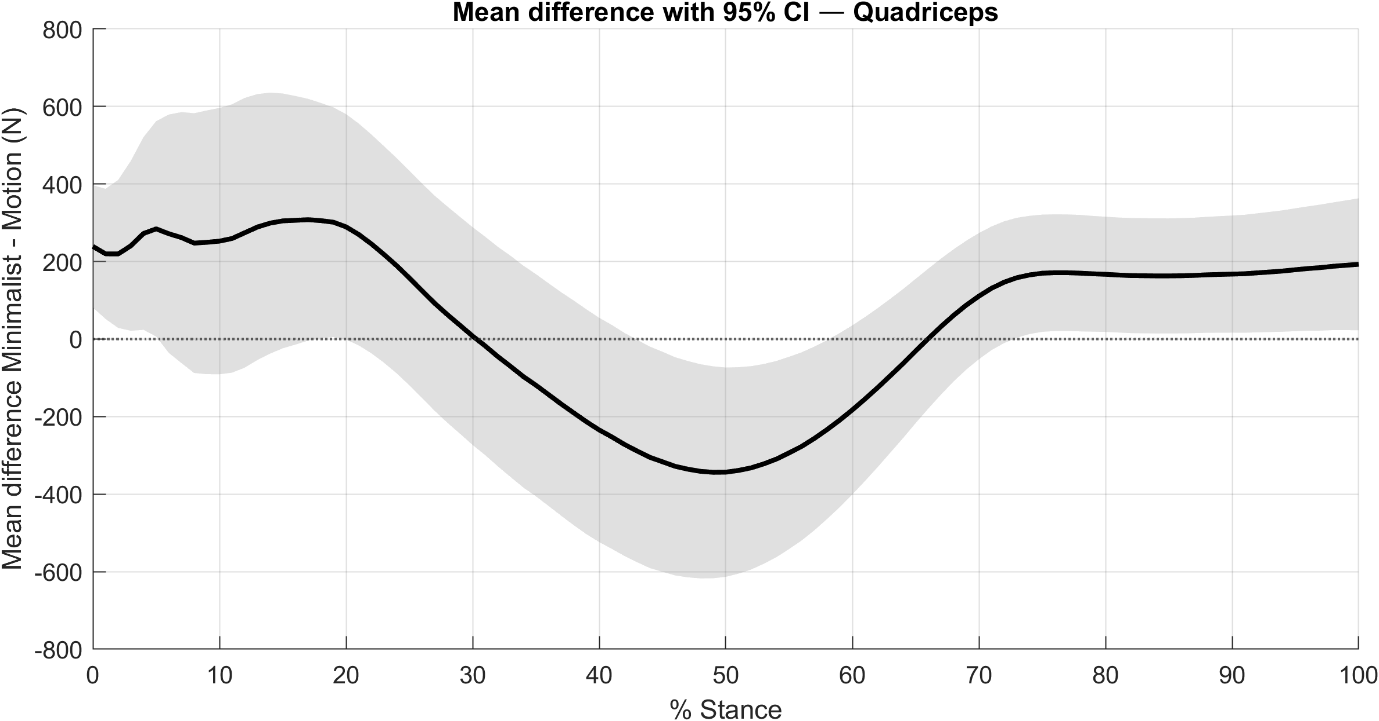
**

Quadriceps t graph (top) and plotted mean difference with 95% confidence interval (CI) (bottom). The mean difference is represented as the mean minimalist force minus the mean motion force.

**
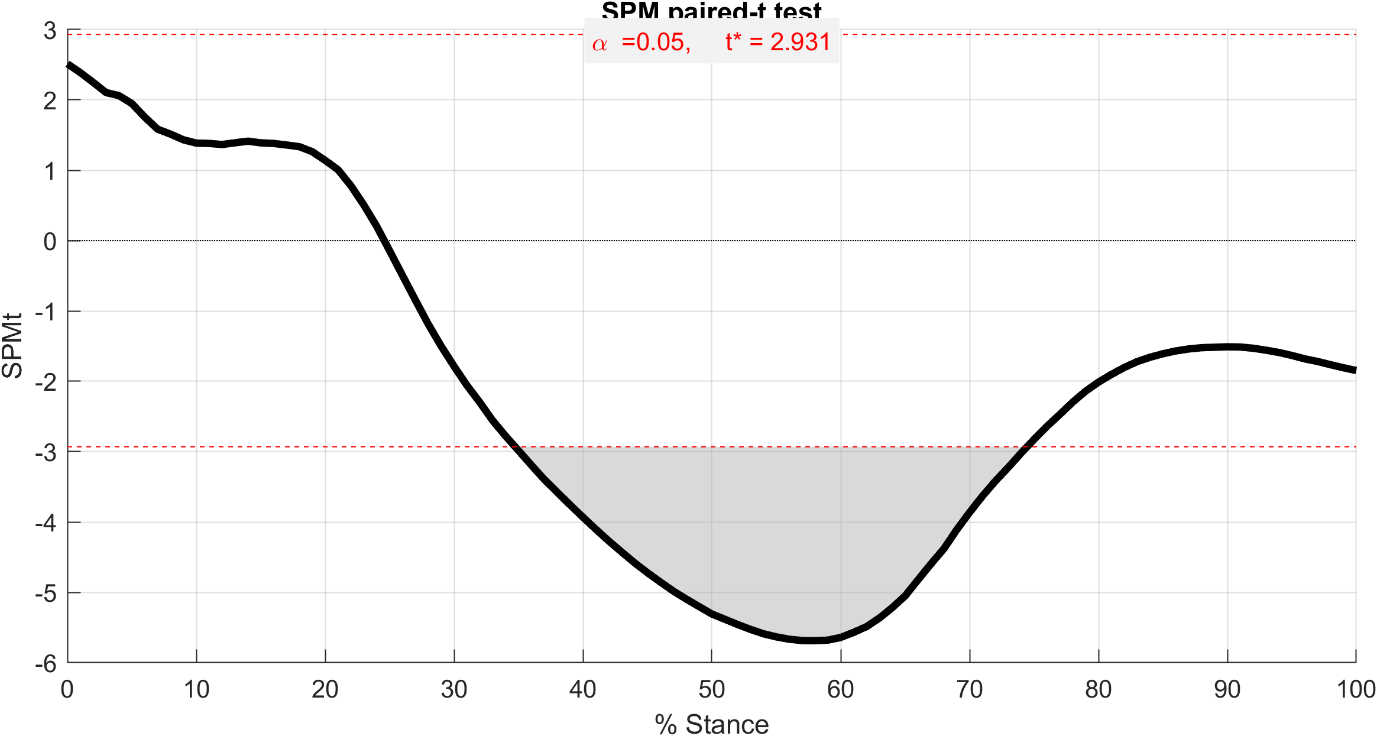
**

**
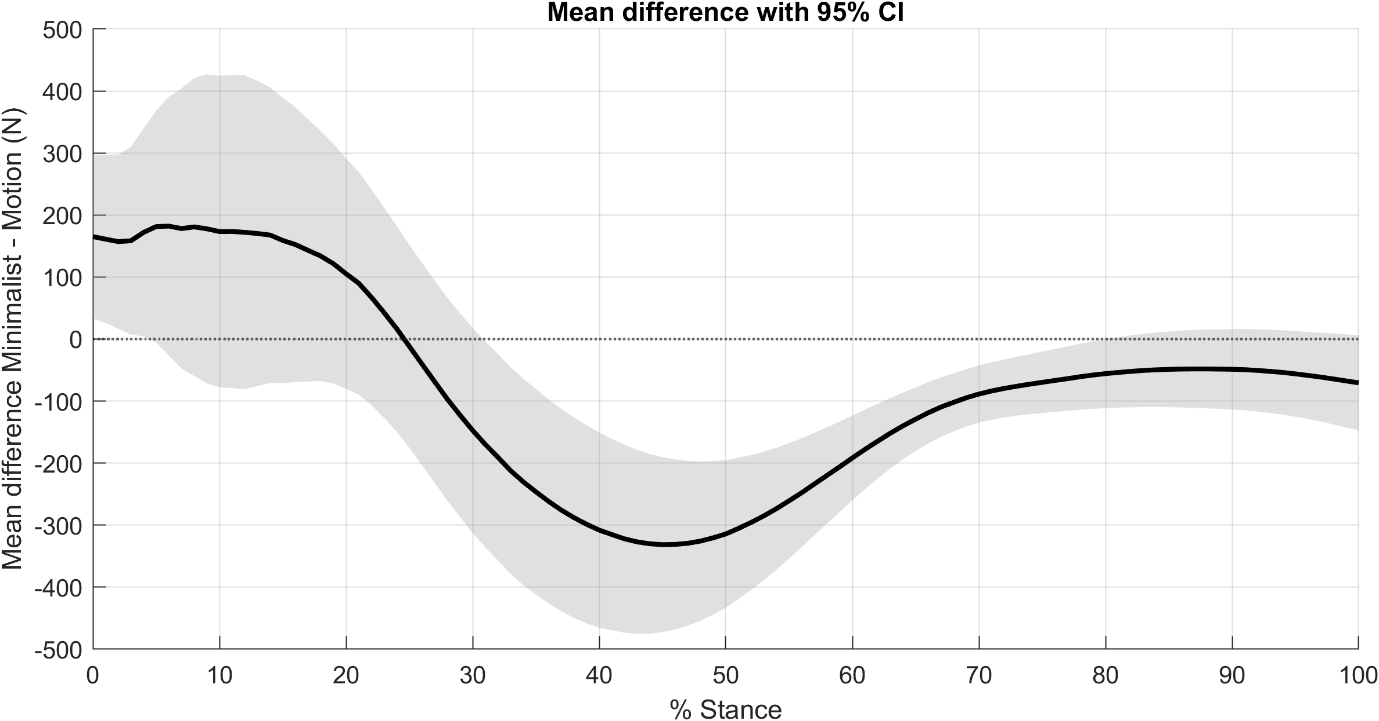
**

Lateral patella force t graph (top) and plotted mean difference with 95% confidence interval (CI) (bottom). The mean difference is represented as the mean minimalist force minus the mean motion force.

**
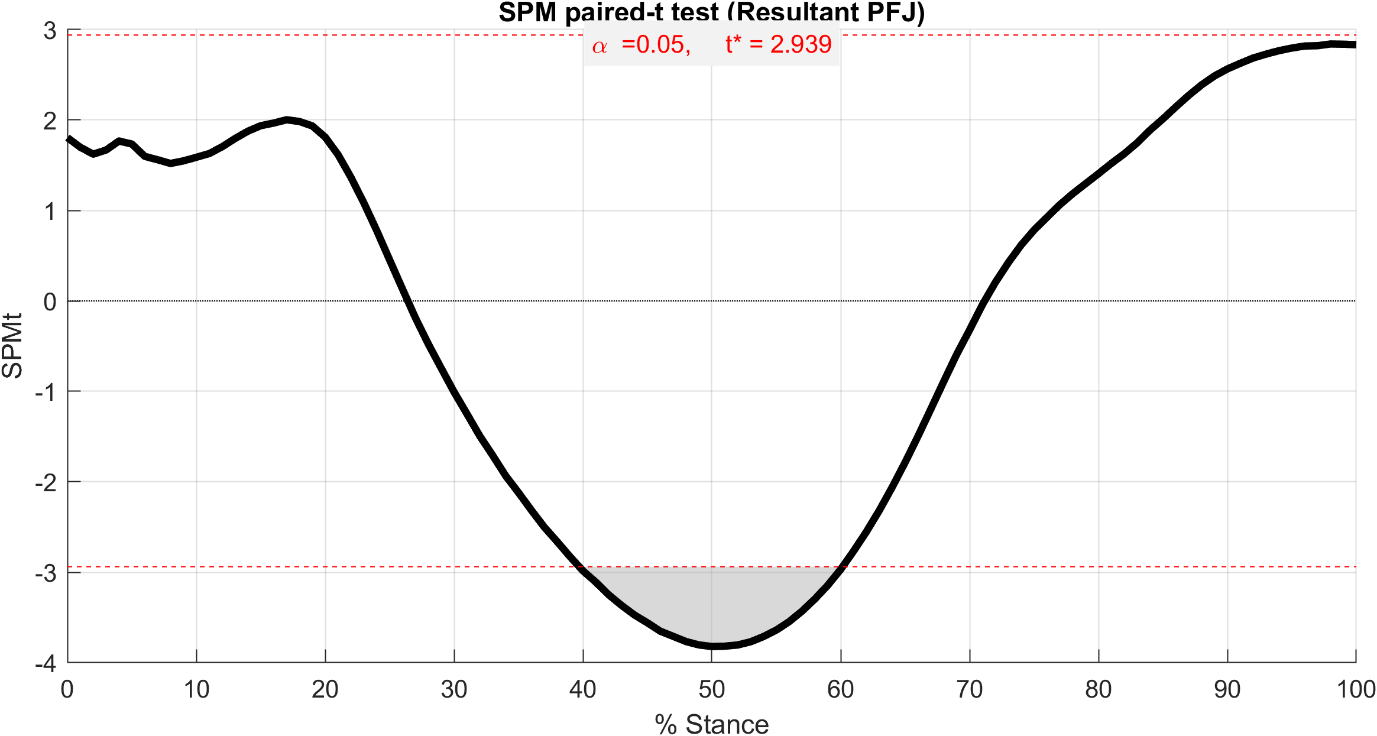

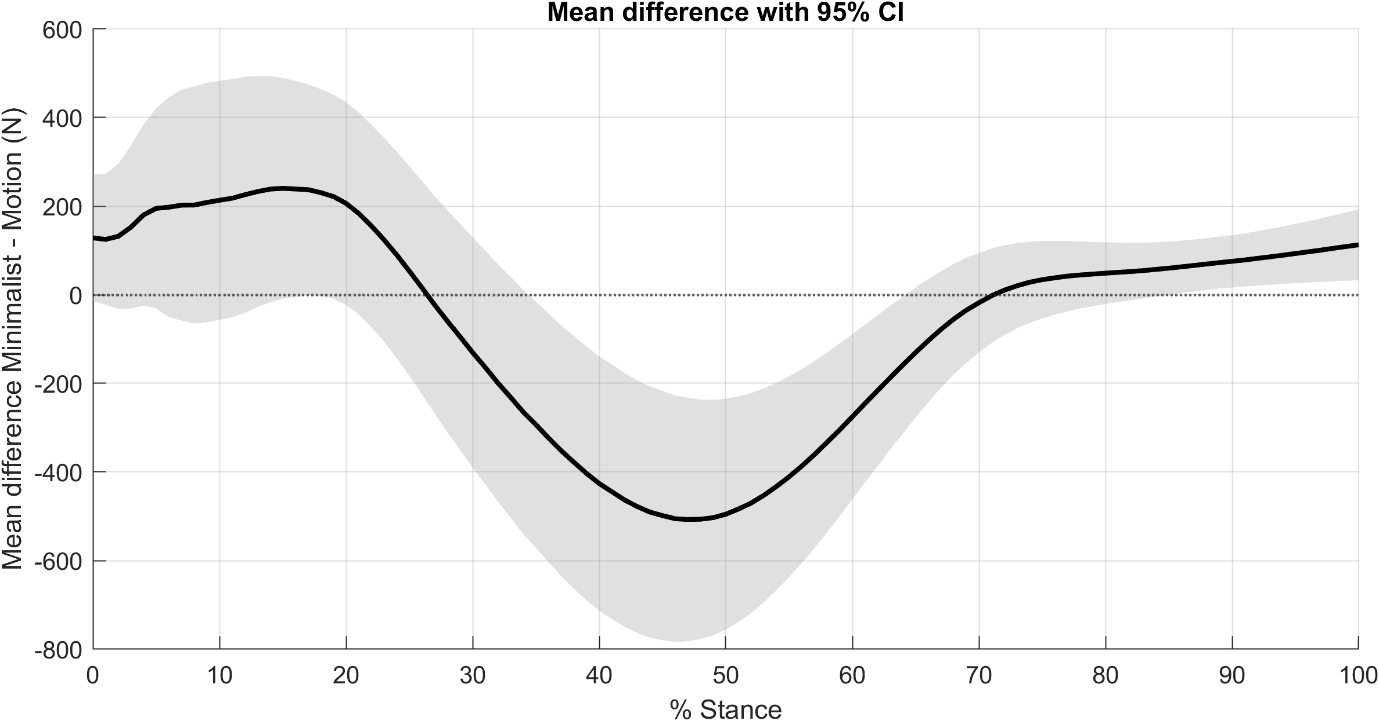
**

Resultant patellofemoral joint force t graph (top) and plotted mean difference with 95% confidence interval (CI) (bottom). The mean difference is represented as the mean minimalist force minus the mean motion force.

**Model Validation**

*Activations*


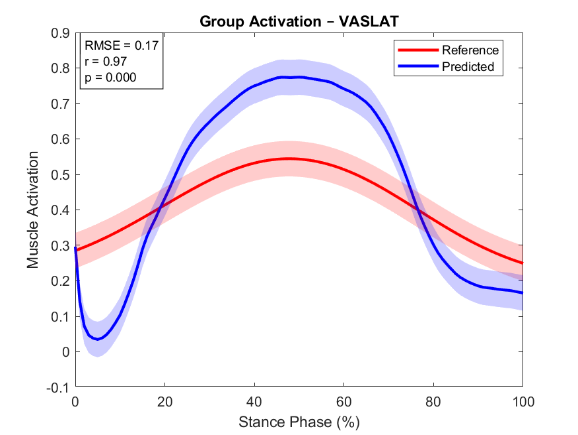

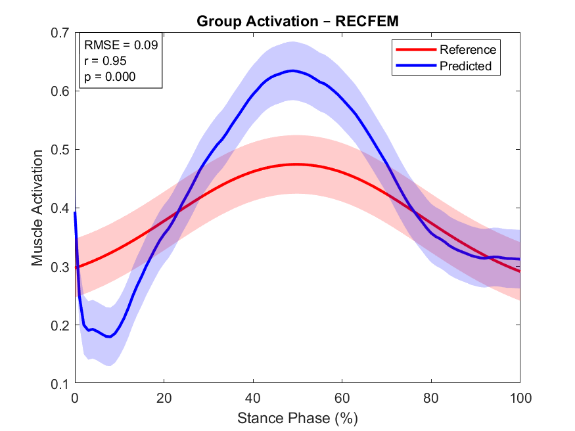

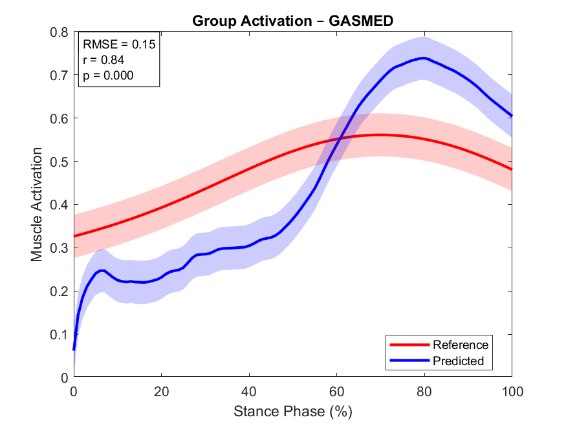

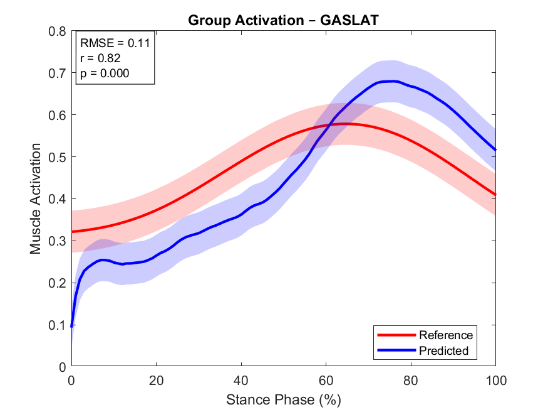

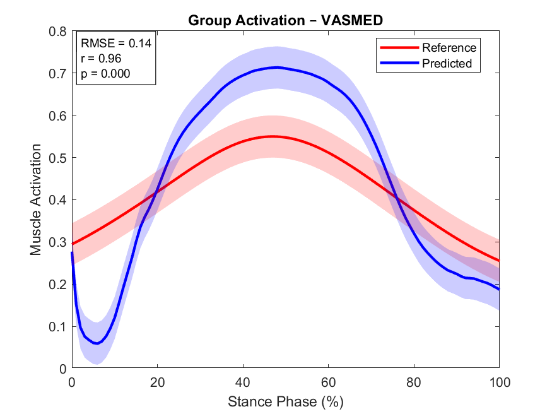


EMG reference (i.e., measured) muscle activation (red) versus model-predicted muscle activation (blue) for the eight muscles of interest during the stance phase of running averaged over the two footwear conditions. Pearson’s correlation coefficient (r) and the root mean square errors (RMSE) are reported for each muscle. The lines represent the mean values of muscle activation level, and the shaded area represents the standard deviation. Zero represents no activation, and one represents maximal activation.

***Torques***


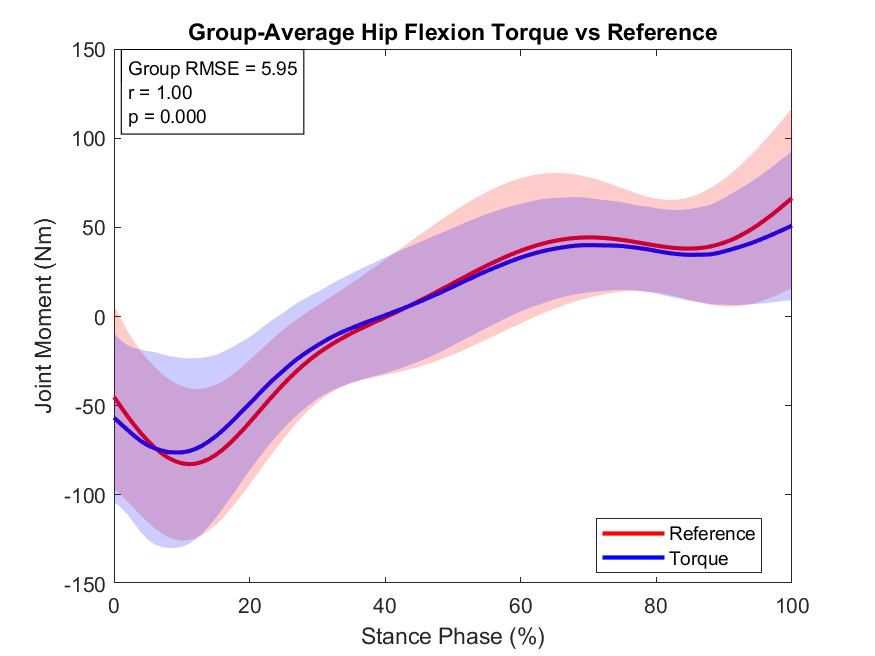

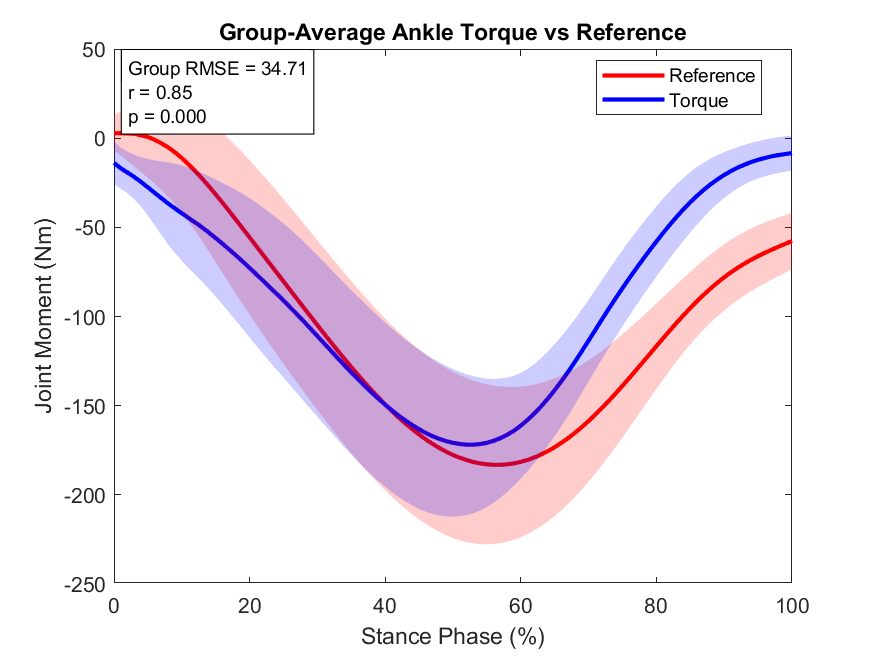

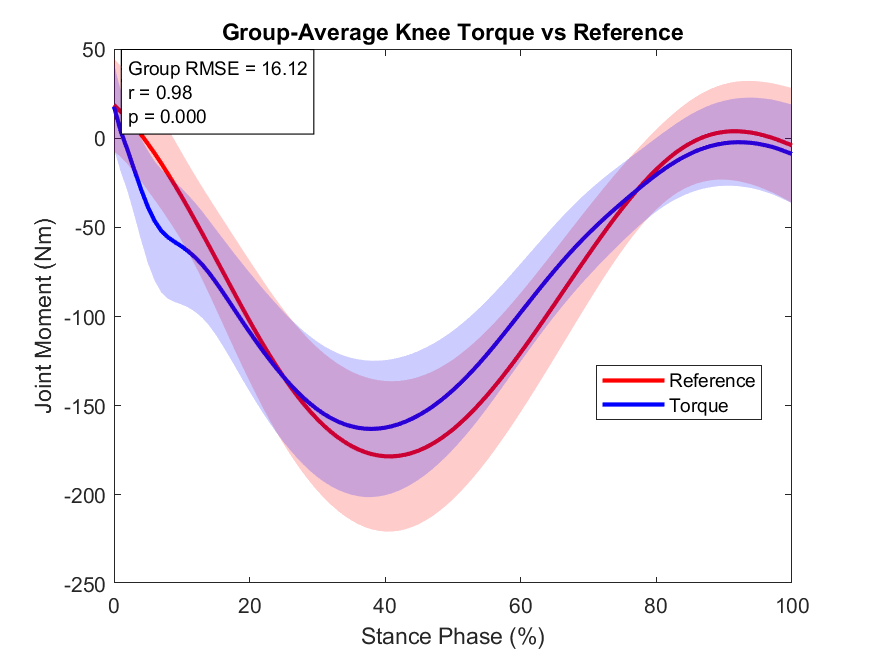


Hip, knee and ankle joint moments (Nm) derived using inverse dynamics (i.e., reference) (red) and predicted (blue) using the model during stance, averaged over the two footwear conditions. Pearson’s correlation coefficient (r) and the root mean square errors (RMSE) are reported for each joint. The lines represent the mean values of joint torque, and the shaded area represents the standard deviation. For knee torque, negative values represent extension, and positive values represent flexion. For ankle torque, negative represents plantar flexion and positive represents dorsiflexion.

**Supplementary table 3: EMG model sensitivity analysis**

| **Model** | **Minimalist vs Motion (95% CI)** | **R²** |
| --- | --- | --- |
| 1. Minimalist only (N) | 0.8 (0.6, 1.0) | 0.68 |
| 2. Minimalist + EMG System covariate (N) | 0.8 (0.6, 0.9) | 0.70 |
| Minimalist vs Motion column = every one newton (N) of force during running in the motion shoe is equivalent to 0.8 and 0.8 newtons in the minimalist shoe in models one and two, respectively; 95% CI = 95% Confidence Interval.  Model 1 = relationship between minimalist and motion peak force  Model 2 = relationship between minimalist and motion peak force with EMG system covariate  Similar coefficients with EMG system added as a covariate indicate no evidence of effect from the different EMG systems. | | |

**Supplementary table 4: Marker placement**

| Position |
| --- |
| C7 spinous process |
| Manubrium |
| PSIS bilaterally |
| Acromion bilaterally |
| ASIS bilaterally |
| Antero-lateral thigh bilaterally |
| Medial and lateral epicondyle of femur |
| Shank cluster (three markers) |
| Thigh cluster (four markers) |
| Head of first metatarsal |
| Head of third metatarsal |
| Head of fifth metatarsal |
| Base of fifth metatarsal |
| Calcaneus just below achillies tendon |
| Medial malleolus |
| Lateral malleolus |

# Supplementary table 5: Report PFP Checklist

Checklist of strongly recommended and recommended items for quantitative patellofemoral pain studies

| **Section 1 – Items Strongly Recommended**  **(Essential)** | | **Reported on page # or N/A** |
| --- | --- | --- |
| ***Demographics*** | |  |
| 1 | Sex or gender of the participants | 11 |
| 2 | Age of the participants | 11 |
| ***Baseline symptoms*** | |  |
| 3 | Symptom duration | 11 |
| 4 | Pain Severity | 11 |
| 5 | Unilateral/bilateral symptoms | N/A |
| ***Outcome measures*** | |  |
| 6 | Condition specific patient-reported outcome | 11 |
| 7 | Pain severity | 11 |
| ***Outcome measure description*** | |  |
| 8 | Describe assessment in adequate detail to allow replication | 8, 9 and supp file |
| ***Reporting study results*** | |  |
| 9 | Mean and standard deviation for parametric data | 12 and 13 |
| 10 | Median and interquartile range for non-parametric data | N/A |
| 11 | Precision of estimate for all inferential statistics (e.g. 95% confidence interval for between group differences) | 12 and 13 |
| **Section 2 – Items Recommended**  **(encouraged but are not required to meet consensus recommendations)** | | **Reported on page # or N/A** |
| ***Demographics*** | |  |
| 12 | Anthropometrics (including body mass and height or body mass index) | 11 |
| 13 | Physical activity levels | 11 |
| 14 | Source/setting/location of participants | 6 |
| 15 | Ethnicity of the participants | N/A |
| ***Baseline symptoms and previous treatment*** | |  |
| 16 | Previous treatment | N/A |
| 17 | Pain location(s) | N/A |
| 18 | Aggravating factors | 7 |
| 19 | History of knee surgery | 7 |
| 20 | Other symptoms, musculoskeletal symptoms, and comorbidities | 7 |
| 21 | Crepitus | N/A |
| 22 | Pain quality | N/A |
| ***Outcome measures*** | |  |
| 23 | Physical activity | N/A |
| 24 | Global rating of change | N/A |
| 25 | Health-related quality of life | N/A |
| 26 | Psychological factors (including self-efficacy, pain-related fear and pain catastrophising) | N/A |
| ***Outcome measure description*** | |  |
| 27 | Provide measurement properties of assessments | 9 |
| 28 | Provide videos and/or images of assessments | Supp file 1 |
| ***Clinical trial methodology*** | |  |
| 29 | Follow recommendations from EQUATOR Network^2^ | N/A |
| 30 | Use existing checklists for interventions, including TIDiER; CERT for exercise interventions; and Toigo and Boutellier for resistance training interventions | N/A |
| 31 | Provide videos and/or images of treatments | N/A |

N/A = not applicable

CERT = Complete Exercise Reporting Template^1^; EQUATOR = Enhancing the QUAlity and Transparency Of health Research^2^; TIDiER = Template of Intervention Description and Replication^3^.

**
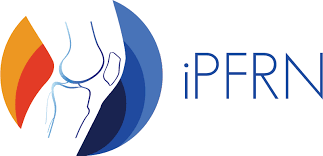
**

**References**

1. Slade SC, Dionne CE, Underwood M*, et al.* Consensus on Exercise Reporting Template (CERT): Explanation and Elaboration Statement. British Journal of Sports Medicine. 2016; 50:1428-1437.
2. <https://www.equator-network.org/>
3. Hoffmann TC, Glasziou PP, Boutron I, Milne R, Perera R, Moher D et al. Better reporting of interventions: template for intervention description and replication (TIDieR) checklist and guide. *British Medical Journal*. 2014; 348:g1687.
4. Toigo M, Boutellier U. New fundamental resistance exercise determinants of molecular and cellular muscle adaptations. European Journal Applied Physioly. 2006, 97(6):643-663. Guidance to Toigo and Boutellier resistance training reporting.
